# Prescription characteristics associated with drug overdose risk among adults prescribed benzodiazepines: a cohort study

**DOI:** 10.1186/s40360-023-00674-x

**Published:** 2023-05-19

**Authors:** Donovan T. Maust, Amy S. B. Bohnert, Julie Strominger, Jason E. Goldstick

**Affiliations:** 1 Injury Prevention Center, University of Michigan, Ann Arbor, MI 48109 USA; 2 Department of Psychiatry, University of Michigan Medical School, Ann Arbor, MI USA; 3 Center for Clinical Management Research, VA Ann Arbor Healthcare System, Ann Arbor, MI USA; 4 Institute for Healthcare Policy and Innovation, University of Michigan, Ann Arbor, MI USA; 5 Department of Anesthesiology, University of Michigan Medical School, Ann Arbor, MI USA; 6 Department of Emergency Medicine, University of Michigan Medical School, Ann Arbor, MI USA

**Keywords:** Benzodiazepine, Cox proportional hazards, Medication possession ratio, Overdose, Poisoning

## Abstract

**Background:**

Drug overdose (OD) deaths in the U.S. continue to rise. After opioids, benzodiazepines (BZD) are the medication most commonly involved in prescription overdoses, yet OD risk factors among those prescribed BZD are not well understood. Our objective was to examine characteristics of BZD, opioid, and other psychotropic prescriptions associated with increased drug OD risk following a BZD prescription.

**Methods:**

We completed a retrospective cohort study using a 20% sample of Medicare beneficiaries with prescription drug coverage. We identified patients with a BZD prescription (“index”) claim between 1 April 2016 and 31 December 2017. In the 6 months pre-index, those without and with BZD claims comprised incident and continuing cohorts, which were split by age (incident < 65 [*n* = 105,737], 65 + [*n* = 385,951]; continuing < 65 [*n* =  240,358], 65 + [*n* = 508,230]). Exposures of interest were: average daily dose and days prescribed of the index BZD; baseline BZD medication possession ratio (MPR) for the continuing cohort; co-prescribed opioids and psychotropics. Our primary outcome was a treated drug OD event (including accidental, intentional, undetermined, or adverse effect) within 30 days of the index BZD, examined using Cox proportional hazards.

**Results:**

Among incident and continuing BZD cohorts, 0.78% and 0.56% experienced an OD event. Compared to 14–30 days, a < 14-day fill corresponded to higher OD risk in incident (< 65 adjusted hazard ratio [aHR] 1.16 [95% CI 1.03–1.31]; 65 + : aHR 1.21 [CI 1.13–1.30]) and continuing (< 65: aHR 1.33 [CI 1.15–1.53]; 65 + : aHR 1.43 [CI 1.30–1.57]) cohorts. Among continuing users, lower baseline exposure (i.e., MPR < 0.5) was associated with increased OD risk for those < 65 (aHR 1.20 [CI 1.06–1.36]); 65 + (aHR 1.12 [CI 1.01–1.24]). Along with opioids, concurrent antipsychotic use and antiepileptic use were associated with elevated risk of OD in all 4 cohorts (e.g., aHRs for the continuing 65 + cohort: opioid, 1.73 [CI 1.58–1.90]; antipsychotic, 1.33 [CI 1.18–1.50]; antiepileptic, 1.18 [1.08–1.30]).

**Conclusions:**

In both the incident and continuing cohorts, patients dispensed fewer days' supply were at increased OD risk; those in the continuing cohort with more limited baseline BZD exposure were also at elevated risk. Concurrent medication exposures including opioids, antipsychotics, and antiepileptics were associated with short-term elevated OD risk.

**Supplementary Information:**

The online version contains supplementary material available at 10.1186/s40360-023-00674-x.

## Background

Drug overdose (OD) deaths in the U.S. recently exceeded 100,000 during a 12-month period for the first time [1]. The contributing role of benzodiazepines (BZD) has received relatively little attention [2, 3], even though they are the second-most common prescription drug involved in OD deaths [4]. The lack of attention to the role of BZD is particularly striking given that, from 1996 to 2013, both the prevalence and total volume of BZD prescriptions increased, while the BZD-related OD death rate increased more than five-fold [5]. While the rate of increase has slowed, in 2020 the U.S. had the highest ever number of BZD-involved OD deaths, at 12,290 [6]. The contribution of prescription BZD to OD is particularly important because, unlike with opioids [7], the vast majority of BZD-involved OD deaths are related to prescribed rather than illicit BZD [8].

Despite the potential risks associated with BZD use, prescriptions in some clinical situations are warranted, such as for patients with treatment-resistant anxiety disorders or refractory epilepsy [9–11]. However, little is known about OD risk among those prescribed BZD to inform and personalize decision-making. While expert opinion and professional guidelines suggest that short-term or intermittent BZD use has a more favorable risk–benefit profile [9, 12, 13], there are few data to support this. Patients with limited BZD exposure may be slower to develop physiological tolerance [14], meaning BZD sedative effects would be more pronounced than with regular consumption. Thus, counter to conventional wisdom, OD risk might be higher in those receiving brief or intermittent BZD prescriptions. And while co-prescribing of BZD with opioids increases risk of respiratory suppression and death—and is now subject to a black box warning from the U.S. Food and Drug Administration [15]—data informing whether other BZD combinations are associated with elevated OD risk are lacking.

To support the safest prescribing possible, clinicians might benefit from additional evidence to inform OD risk among their patients. In this analysis, we used a national sample of Medicare beneficiaries to identify characteristics associated with risk of a treated OD event within 30 days following a BZD prescription, focusing on the BZD and other prescription medications, specifically opioids and psychotropics. We hypothesized that lower BZD exposure would be associated with higher risk, while, along with opioids, co-prescriptions of antipsychotics and antiepileptics would also be associated with increased OD risk.

## Methods

### Study population

For this retrospective cohort study, we began with a 20% random sample of Medicare beneficiaries (Medicare is national, government-sponsored insurance in the U.S. for those ≥ 65 as well as permanently disabled individuals) with ≥ 6 months of continuous fee-for-service and Part D prescription drug coverage between October 2015 and December 2017 (Figure S1). The 20% Medicare sample is a random 20% sample of all Medicare beneficiaries across the U.S. that is made available to researchers through a data use agreement. The 20% sample is provided in lieu of complete data due to the size of the full data; however, because the 20% sample is random, it is representative of the overall Medicare population [16].

Because we were interested in OD risk among those prescribed a BZD, study entry was determined using dates of BZD fills: We identified all BZD fills (“treatment episode” hereafter) during periods of continuous coverage that occurred between April 1, 2016 and December 31, 2017. We limited treatment episodes to those preceded by 6 months of continuous coverage (“baseline”), which we used to derive baseline cohort characteristics. We excluded treatment episodes where the baseline included a treatment OD event (see *Outcome* section for how these were identified). Given the 6-month baseline requirement, the first possible treatment episode started on April 1, 2016, with a baseline period from October 1, 2015 (i.e., the start of *International Classification of Diseases, 10*^*th*^* Revision–Clinical Modification* [*ICD-10-CM*] use in the U.S.) through March 31, 2016.

Treatment episodes were classified as incident or continuing based on whether the individual had prior BZD fills during the 6-month baseline. If there were no BZD fills during baseline, the treatment episode was considered incident; if there was at least 1 BZD fill during baseline, the episode was considered continuing.

Incident and continuing cohorts were then constructed using the identified treatment episodes. The incident cohort included each individual with an incident treatment episode (i.e., a BZD fill on or after 1 April 2016 with no fill during the prior 6 months); if a beneficiary had multiple eligible incident treatment episodes, only the first was included. For each cohort member, their index date was the BZD fill date that began the treatment episode; the BZD dispensed at that time was the index BZD prescription. We identified the continuing cohort by applying these same steps to continuing treatment episodes (i.e., where there was at least one BZD fill during the 6-month baseline).

### Outcome

Each cohort was followed from the index date (i.e., receipt of BZD prescription) until the earliest of the following: treated OD event, death, loss of coverage or start of Medicare Advantage, or 30 days after the index date. We included all OD events—not just those specifically attributed to BZD—by identifying drug OD visits per surveillance recommendations from the U.S. Centers for Disease Control and Prevention (CDC) using *ICD-10-CM* codes for overdose by drugs, medicaments, and biological substances (i.e., T36-T50) [17]. In addition, following Bushnell et al., we included encounters for adverse effects (e.g., T42.4X5) [3]. CDC guidance suggests that *ICD-10-CM* codes for poisoning or OD are for “improper use” of medication, whereas “adverse effect” is for medication that has been “properly administered” (i.e., as prescribed)[18]: Given that patients may experience adverse effects when taking their medications as prescribed—particularly older adults—we thought it was important to capture such events.

### Exposures

Our primary exposures of interest were characteristics of the index BZD as well as prescriptions of opioid and other psychotropic medications (Table S1). For the index BZD, we derived average daily dose (standardized as < 1, 1–1.99, or 2 + lorazepam-equivalent [lor-eq] mg/day hereafter [19]) and days’ supply (< 14, 14–30, or 30 + days). For the continuing BZD cohort, we also measured the baseline BZD medication possession ratio (MPR), computed as the sum of BZD days’ supply during baseline divided by 180 days (i.e., the 6-month baseline; Figure S2), categorized as < 0.5, 0.5–1, or > 1. The BZD exposure variables were categorized based on clinically meaningful cut-points similar to prior analyses [20, 21].

For the other medications of interest, we used prescription fill dates and days’ supply during the 6-month baseline to classify each cohort member, on the index date, as a current (exposure that covered the index date), former (exposure during baseline that did not include the index date), or non-user (no days of exposure during baseline) of: antidepressants, antiepileptics, antipsychotics, opioids, and non-benzodiazepine benzodiazepine receptor agonists (i.e., ‘Z-drugs’). For example, a cohort member with a 30-day antidepressant prescription that was filled 45 days before the index date and no other antidepressant fills was considered a “former” antidepressant user.

### Other characteristics

We controlled for factors potentially associated with the exposures of interest and OD, including age, sex, race/ethnicity, low-income subsidy, rurality, and clinical characteristics. Race/ethnicity was derived using the Research Triangle Institute race variable from the Medicare Master Beneficiary Summary File. Low-income subsidy was considered present if, during baseline, a beneficiary had ≥ 1 month eligible for or enrolled in the Part D low-income subsidy. Rurality was derived using Rural–Urban Continuum Codes.

We used baseline encounters to identify the presence of substance use disorders, personality disorders, and the Elixhauser comorbidity index [22]—a count of 30 conditions—to capture overall burden of medical comorbidity. We did not account for additional psychiatric disorders because, were they present, the analysis already included medications that would be prescribed as exposures of interest. Finally, we included an indicator for frailty, which is an age-related condition of decline in physiological function and increased susceptibility to stressors [23] associated with adverse outcomes among older adults including mortality and falls. For the incident and continuing 65 + cohorts, we measured frailty using a claims-based frailty index, which is a weighted scale including medical conditions and durable medical equipment (e.g., walking aids) [24]. The scale of the frailty index is 0 to 1, which we dichotomized as not frail (< 0.2) and frail (≥ 0.2) [25].

### Statistical analysis

We decided a priori to conduct analyses stratified by age group given that characteristics associated with an OD event may differ for younger, disability-eligible Medicare beneficiaries versus age-eligible counterparts. Therefore, we split the incident and continuing cohorts based on beneficiary age on the index date, yielding four analytic cohorts (incident < 65 and 65 + ; continuing < 65 and 65 +). For each cohort, we summarized beneficiary characteristics, the index BZD prescribed, and the specific type of OD injury diagnosis.

For each cohort, we plotted Kaplan–Meier survival curves within each cohort, stratified by BZD exposure variables of interest (MPR, days’ supply, average daily dose). We used log-rank tests to examine if survival functions were equal across levels of the given exposure variable (e.g., MPR). We then fit Cox proportional hazards models for each cohort to examine factors associated with an OD event following the index date (i.e., BZD prescription fill). We determined if the assumption of proportional hazards was met by examining plots of Schoenfeld residuals versus time. To account for those lost to follow up due to death or loss of coverage (i.e., artificial censoring), we used inverse probability weighting to recover estimates consistent with a population without artificial censoring [26]. Specifically, we: 1) fit a binary logistic regression model where the outcome was not being censored due to death or loss of coverage; 2) used the predicted values from that model to construct weights as 1 / [predicted probability of not being censored due to death or loss of coverage]; and 3) applied those weights to a Cox regression model using only those who were not artificially censored. Thus, estimates from the weighted Cox model represent those that would have been obtained from a study with no artificial censoring. Across all cohorts, weights ranged from 1.001 to 60.88; the 99^th^ percentile was 1.80. Because the prevalence of missing data was low and limited to race/ethnicity (i.e., 0.8 to 1.4% across the 4 cohorts), complete case analysis was used. The significance level was set at 0.05 and all tests were two sided.

For the utility of future researchers designing similar studies, we briefly describe the justification for the study design. On the basis of prior work with the 20% Medicare sample, the prevalence of benzodiazepine use, and extent of incident use [27, 28], we conservatively estimated an incident cohort of approximately *N* = 205,200 patients. Due to the novelty of this work, we did not, a priori, know the base rate of overdose specifically among BZD users, and thus this was a free parameter in our power calculations. Based on our anticipated sample size, were the base rate of overdose either 0.1%, 0.5%, or 1.0%, we would be sufficiently powered (> 80% power) to detect absolute risk differences of 0.08%, 0.17%, and 0.22%, respectively. In terms of Cohen’s H, a standard effect size for quantifying differences between proportions (small: 0.2; medium: 0.5; large: 0.8) [29], each of these differences correspond to roughly H = 0.02, a very small effect size. Cast in terms of odds ratios, which are likely more familiar, the risk differences stated above would translate to values of approximately 1.82, 1.34, and 1.22, respectively—all small effect sizes [30].

We conducted analyses using SAS 9.4 and created figures in R version 4.1.0 using the “ggplot2” package. Analyses were approved by the Michigan Medicine IRB; informed consent was waived for this analysis of observational data.

## Results

### Incident cohorts

The incident < 65 cohort included 105,737 adults newly prescribed a BZD; 60.2% were female and 71.8% were non-Hispanic white (Table 1). The incident 65 + cohort included 385,951 adults; 69.1% were female and 87.2% were non-Hispanic white. Of the non-BZD concurrent medication exposures of interest, antidepressants and antiepileptics were most common for those < 65 (46.8% and 36.7%, respectively) and antidepressants and opioids for those 65 + (34.4% and 20.1%). The mean average daily BZD dose for the < 65 and 65 + cohorts was 1.9 (standard deviation [SD] 1.8) and 1.5 (SD 1.5) lor-eq mg/day, while days’ supply was 19.1 (SD 16.4) and 23.0 (SD 21.3). Lorazepam was most-commonly prescribed to those < 65 (31.3%; Table 2), while alprazolam was most-commonly prescribed to those 65 + (35.0%).

**Table 1:** Characteristics of Incident and Continuing Benzodiazepine Cohorts, Stratified by Age

**Characteristic, n (%)**	**Benzodiazepine Use**			
	**Incident**		**Continuing**	
	** < 65** ***N* = 105,737**^**a**^	**65 + ** ***N* = 385,951**^**b**^	** < 65** ***N* = 240,358**^**c**^	**65 + ** ***N* = 508,230**^**d**^
*Sociodemographics*				
Sex				
Male	42,092 (39.8)	119,263 (30.9)	93,756 (39.0)	147,147 (29.0)
Female	63,645 (60.2)	266,688 (69.1)	146,602 (61.0)	361,083 (71.0)
Age				
< 45	27,107 (25.6)	-	56,205 (23.4)	-
45–64	78,630 (74.4)	-	184,153 (76.6)	-
65–74	-	202,492 (52.5)	-	274,193 (54.0)
75–84	-	122,367 (31.7)	-	156,586 (30.8)
85 +	-	61,092 (15.8)	-	77,451 (15.2)
Race^e^				
Non-Hispanic White	75,898 (71.8)	336,567 (87.2)	188,167 (78.3)	446,672 (87.9)
Non-Hispanic Black	17,067 (16.1)	18,941 (4.9)	28,135 (11.7)	23,455 (4.6)
Hispanic	9,754 (9.2)	19,638 (5.1)	18,517 (7.7)	26,839 (5.3)
Asian/Pacific Islander	1,270 (1.2)	7,496 (1.9)	2,195 (0.9)	7,282 (1.4)
Other	1,748 (1.7)	3,309 (0.9)	3,344 (1.4)	3,982 (0.8)
Low-income subsidy^f^				
No	18,372 (17.4)	299,003 (77.5)	41,594 (17.3)	362,658 (71.4)
Yes	87,365 (82.6)	86,948 (22.5)	198,764 (82.7)	145,572 (28.6)
Rurality^g^				
Urban	90,107 (85.2)	336,949 (87.3)	202,623 (84.3)	436,403 (85.9)
Rural	15,630 (14.8)	49,002 (12.7)	37,735 (15.7)	71,827 (14.1)
*Clinical Characteristics*				
Frailty				
Not frail	-	249,972 (64.8)	-	318,165 (62.6)
Frail	-	135,979 (35.2)	-	190,065 (37.4)
Elixhauser				
0–1	37,181 (35.2)	105,604 (27.4)	85,786 (35.7)	130,382 (25.7)
2	18,076 (17.1)	67,376 (17.5)	44,260 (18.4)	93,588 (18.4)
3	14,621 (13.8)	58,547 (15.2)	34,635 (14.4)	81,700 (16.1)
4	10,732 (10.1)	45,316 (11.7)	25,061 (10.4)	62,874 (12.4)
5	7,670 (7.3)	33,589 (8.7)	17,355 (7.2)	45,717 (9.0)
6	5,391 (5.1)	23,783 (6.2)	11,637 (4.8)	31,835 (6.3)
7	3,861 (3.7)	16,765 (4.3)	7,639 (3.2)	21,643 (4.3)
8	2,677 (2.5)	11,934 (3.1)	5,036 (2.1)	14,684 (2.9)
9 +	5,528 (5.2)	23,037 (6.0)	8,949 (3.7)	25,807 (5.1)
Substance Use Disorder	31,593 (29.9)	28,509 (7.4)	73,948 (30.8)	47,401 (9.3)
Personality Disorder	2,851 (2.7)	1,091 (0.3)	7,424 (3.1)	2,415 (0.5)
Bipolar Disorder	15,034 (14.2)	6,826 (1.8)	43,626 (18.2)	16,190 (3.2)
Lithium use	2,690 (2.5)	941 (0.2)	7,728 (3.2)	2,080 (0.4)
*BZD Characteristics*				
Medication possession ratio				
< 0.5	-	-	105,109 (43.7)	322,688 (63.5)
0.5–1	-	-	118,136 (49.2)	169,230 (33.3)
> 1	-	-	17,113 (7.1)	16,312 (3.2)
Index average daily dose, mg				
< 1	20,328 (19.2)	113,194 (29.3)	23,421 (9.7)	110,961 (21.8)
1–1.99	40,980 (38.8)	167,657 (43.4)	60,222 (25.1)	197,115 (38.8)
2 +	44,429 (42.0)	105,100 (27.2)	156,715 (65.2)	200,154 (39.4)
Index supply, days				
< 14	44,866 (42.4)	147,779 (38.3)	24,135 (10.0)	60,447 (11.9)
14–30	56,981 (53.9)	204,690 (53.0)	198,259 (82.5)	369,013 (72.6)
31 +	3,890 (3.7)	33,482 (8.7)	17,964 (7.5)	78,770 (15.5)
*Other Medication Use*				
Antidepressant				
Never	45,680 (43.2)	226,769 (58.8)	79,125 (32.9)	241,650 (47.5)
Former	10,550 (10.0)	26,533 (6.9)	25,973 (10.8)	42,380 (8.3)
Current	49,507 (46.8)	132,649 (34.4)	135,260 (56.3)	224,200 (44.1)
Antiepileptics				
Never	57,040 (53.9)	307,492 (79.7)	119,834 (49.9)	385,166 (75.8)
Former	9,861 (9.3)	20,935 (5.4)	23,527 (9.8)	30,577 (6.0)
Current	38,836 (36.7)	57,524 (14.9)	96,997 (40.4)	92,487 (18.2)
Antipsychotics				
Never	78,103 (73.9)	353,526 (91.6)	158,759 (66.1)	448,824 (88.3)
Former	5,110 (4.8)	8,738 (2.3)	15,097 (6.3)	15,689 (3.1)
Current	22,524 (21.3)	23,687 (6.1)	66,502 (27.7)	43,717 (8.6)
Opioids				
Never	49,397 (46.7)	240,380 (62.3)	93,089 (38.7)	286,793 (56.4)
Former	19,544 (18.5)	68,000 (17.6)	45,933 (19.1)	107,692 (21.2)
Current	36,796 (34.8)	77,571 (20.1)	101,336 (42.2)	113,745 (22.4)
Z-drugs				
Never	95,512 (90.3)	358,797 (93.0)	208,111 (86.6)	463,760 (91.3)
Former	3,146 (3.0)	11,041 (2.9)	9,254 (3.9)	19,174 (3.8)
Current	7,079 (6.7)	16,113 (4.2)	22,993 (9.6)	25,296 (5.0)

BZD, benzodiazepine; SD, standard deviation

^a^1.0% had treated overdose event; 94.6% censored at 30d after index, 3.9% for loss of coverage, 0.5% for death

^b^0.7% had treated overdose event; 93.2% censored at 30d after index, 3.4% for loss of coverage, 2.6% for death

^c^0.7% had treated overdose event; 97.2% censored at 30d after index, 1.9% for loss of coverage, 0.2% for death

^d^0.5% had treated overdose event; 97.1% censored at 30d after index, 1.7% for loss of coverage, 0.8% for death

^e^Derived using the Research Triangle Institute race variable; race groups are mutually exclusive

^f^Considered present if a given beneficiary was enrolled or eligible in the Part D low-income subsidy for at least one month during the 6-month baseline period

^g^Derived using beneficiary state and county codes and Rural–Urban Continuum Codes

**Table 2:** Index Benzodiazepine Prescribed to Incident and Continuing Benzodiazepine Users by Age

**Medication, n (%)**^**a**^	**Incident**		**Continuing**	
	** < 65** ***N* = 105,737**	**65 + ** ***N* = 385,951**	** < 65** ***N* = 240,358**	**65 + ** ***N* = 508,230**
Alprazolam	24,559 (23.2)	135,082 (35.0)	73,624 (30.6)	185,634 (36.5)
Lorazepam	33,086 (31.3)	126,578 (32.8)	52,716 (21.9)	148,040 (29.1)
Clonazepam	18,715 (17.7)	33,977 (8.8)	72,094 (30)	84,809 (16.7)
Diazepam	24,228 (22.9)	62,224 (16.1)	32,879 (13.7)	47,370 (9.3)
Temazepam	4,367 (4.1)	23,530 (6.1)	9,623 (4.0)	36,087 (7.1)
Clorazepate	237 (0.2)	2,043 (0.5)	831 (0.3)	4,523 (0.9)
Chlordiazepoxide	466 (0.4)	1,732 (0.4)	443 (0.2)	2,247 (0.4)
Triazolam	237 (0.2)	1,280 (0.3)	342 (0.1)	1,470 (0.3)
Oxazepam	61 (0.1)	352 (0.1)	180 (0.1)	1,006 (0.2)
Flurazepam	24 (0)	201 (0.1)	93 (0)	323 (0.1)

*BZD* Benzodiazepine

^a^List includes the top 10 index BZD prescribed to the continuing 65 + cohort (i.e., the largest group) in descending order. Percentages in a given cohort may sum to > 100% because some beneficiaries filled > 1 index BZD prescription. Estazolam, midazolam, and clobazam each accounted for < 0.1% of BZD fills to the continuing 65 + cohort and are not shown

**Fig. 1:**
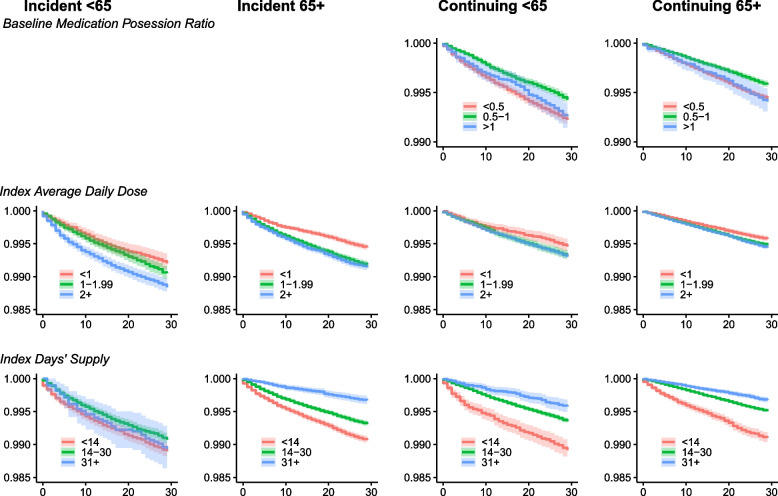
Kaplan Meier Survival Curves of Overdose Events Among Incident and Continuing BZD Users by Age and BZD Exposures. MPR, medication possession ratio; BZD, benzodiazepine. For each panel, the y-axis presents the survival probability and x-axis is days from index BZD prescription. MPR log-rank (LR) test *p*-values: continuing cohorts < 65 (< .0001) and 65 + (< .0001). Average daily dose LR test *p*-values: incident cohorts < 65 (< .0001) and 65 + (< .0001); continuing cohorts < 65 (.03) and 65 + (< .0001). Days’ supply LR test *p*-values: incident cohorts < 65 (.02) and 65 + (< .0001); continuing cohorts < 65 (< .0001) and 65 + (< .0001)

Within 30d of the index BZD, 0.78% of the incident cohort overall experienced a treated OD event (1.0% and 0.7% of those < 65 and 65 +); the events were attributed to BZD among 16.4% and 9.7% of the < 65 and 65 + incident cohorts, respectively (Table S2). Figure 1 presents the Kaplan Meier survival distributions based on index BZD exposure. For both age groups, there appeared to be an inverse relationship between average daily dose and probability of survival (e.g., lowest for those prescribed 2 + lor-eq mg/day (log-rank [LR] test < 65: *p* < 0.0001; 65 + : *p* < 0.0001). For both age groups, those prescribed < 14 days exhibited the lowest survival probability (LR test < 65: *p* = 0.02; 65 + : *p* < 0.0001).

**Fig. 2:**
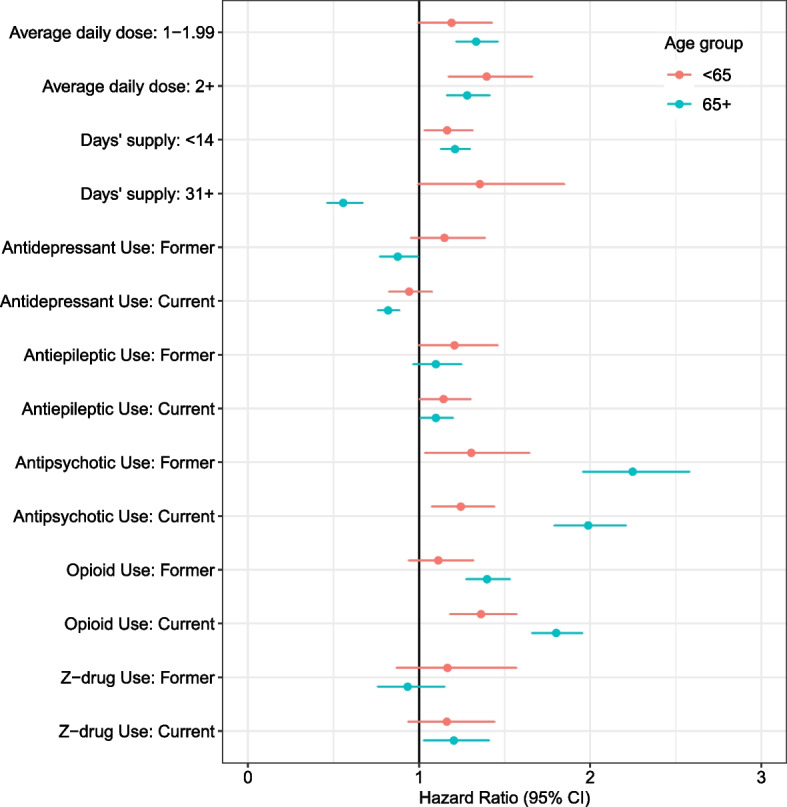
Cox Proportional Hazards Model of Overdose Event Following Incident Benzodiazepine Use Stratified by Age: Benzodiazepine and Other Prescription Medication Characteristics^a^. CI, confidence interval; BZD, benzodiazepine. ^a^ Adjusted for sociodemographic characteristics, Elixhauser comorbidity index, frailty (among the 65 + age group), and seasonality (full model presented in Table S2). Reference groups are as follows: Average daily dose (< 1 lorazepam-equivalent mg/day); Days’ supply (14-30 days); specific medication classes (never use)

Figure 2 presents associations between medication exposures of interest and adjusted hazards of an OD event within 30 days (unadjusted and fully adjusted results in Tables S3 and S4). In both cohorts, those prescribed 2 + lor-eq mg/day had a higher risk of OD compared to those prescribed < 1 mg/day (< 65: adjusted hazard ratio [aHR] 1.40 [95% CI 1.17–1.66]; 65 + : aHR 1.28 [CI 1.16–1.41]). For both those < 65 and 65 + , compared to a 14–30 day supply, < 14 days’ supply was associated with increased risk of OD event (< 65: aHR 1.16 [CI 1.03–1.31]; 65 + : aHR 1.21 [CI 1.13–1.30]). Among co-prescribed medications for those prescribed an incident BZD, current antiepileptic, antipsychotic, and opioid use were all associated with increased risk of OD for both < 65 and 65 + cohorts; current Z-drug use was associated with OD for the 65 + cohort.

### Continuing cohorts

The continuing < 65 cohort included 240,358 adults; 61.0% were female and 78.3% were non-Hispanic white (Table 1). The continuing 65 + cohort include 508,230 adults; 71.0% were female and 87.9% were non-Hispanic white. Of the other current medication exposures of interest for both age cohorts, antidepressants and opioids were most common (< 65: 56.3% and 42.2%, respectively; 65 + : 44.1% and 22.4%). The mean average daily BZD dose for the < 65 and 65 + cohorts was 3.2 (standard deviation [SD] 2.9) and 1.9 (SD 1.7) lor-eq mg/day while days’ supply was 29.3 (SD 15.1) and 33.8 (SD 22.9), respectively. Alprazolam was most commonly prescribed to both age groups (< 65: 30.6%; 65 + : 36.5%; Table 2).

Within 30d of the index BZD, 0.56% of the continuing cohort overall experienced a treated OD event (0.7% and 0.5% of those < 65 and 65 +). The treated OD events were attributed to BZD among 18.2% and 9.0% of the < 65 and 65 + continuing cohorts, respectively (Table S2). Figure 1 presents the Kaplan Meier survival distributions based on index BZD exposure. Among both the < 65 and 65 + cohorts, the middle medication possession ratio (MPR 0.5–1) group—those with a medication supply that ranged from half of to all days during the 6-month baseline—exhibited the highest survival probability (LR test p < 0.0001 for both), as did those prescribed < 1 lor-eq mg/day (LR test, < 65: *p* = 0.03; 65 + : *p* < 0.0001). There was an inverse relationship between days’ supply of the index prescription fill and survival probability, with survival lowest for those prescribed < 14 days (LR test *p* < 0.0001 for < 65 and 65 +).

**Fig. 3:**
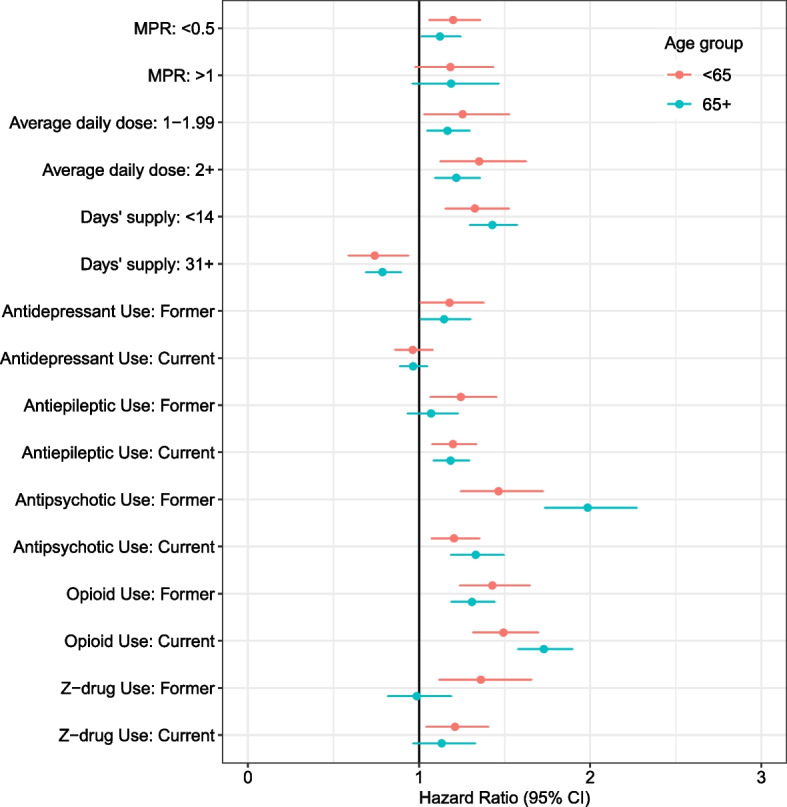
Cox Proportional Hazards Model of Overdose Event Following Continuing Benzodiazepine Use Stratified by Age: Benzodiazepine and Other Prescription Medication Characteristics^a^. MPR, medication possession ratio; CI, confidence interval; BZD, benzodiazepine. ^a^ Adjusted for sociodemographic characteristics, Elixhauser comorbidity index, frailty (among the 65 + age group), and seasonality (full model presented in Table S2). Reference groups are as follows: MPR (0.5-1.0); Average daily dose (< 1 lorazepam-equivalent mg/day); Days’ supply (14-30 days); specific medication classes (never use)

Figure 3 presents associations between medication exposures of interest and adjusted hazards of an OD event within 30 days among continuing users (full results presented in Table S4). In both age groups, those prescribed 2 + lor-eq mg/day had a higher risk of OD compared to those prescribed < 1 mg/day (< 65: aHR 1.35 [CI 1.12–1.63]; 65 + : aHR 1.22 [CI 1.09–1.36]). For both age groups, a smaller index prescription was associated with higher OD event risk (e.g., for < 14d relative to 14-30d, < 65: aHR 1.33 [CI 1.15–1.53]; 65 + : aHR 1.43 [CI 1.30–1.57]). Among continuing users, lower baseline exposure (i.e., MPR < 0.5) was associated with increased OD risk for those < 65 (aHR 1.20 [CI 1.06–1.36]) and those 65 + (aHR 1.12 [CI 1.01–1.24]). Among co-prescribed medications, current antiepileptic, antipsychotic, and opioid use were associated with increased risk of OD event for both age cohorts.

## Discussion

In this large national study of disability- and age-eligible Medicare beneficiaries with Part D prescription drug coverage, we found that, within 30 days of an index prescription, 0.78% of those with an incident BZD prescription and 0.56% of those with ongoing treatment experienced a treated OD event. For both incident and continuing users, the percentage of those who experienced an OD was slightly higher among the younger cohorts. Features of the prescribed BZD were associated with increased risk for all cohorts, including a smaller quantity of BZD prescribed and a higher average daily dose, while, among continuing users, those with lower baseline exposure were at increased risk. Finally, concurrent opioid, antipsychotic, and epileptic prescriptions were associated with increased risk of OD for all cohorts. While these OD deaths are not attributable to BZD alone, factors associated with OD risk among those prescribed BZD are critical to understand given how widely prescribed BZD are [31] and their well-established association with increased OD risk [32–35].

To our knowledge, this is the first analysis to specifically examine OD risk in a cohort of patients prescribed BZDs. We sought to examine the association between a treated OD event and characteristics of the prescribed BZD, including the baseline pattern of BZD use among continuing users. As hypothesized, lower levels of exposure—both the lowest medication possession ratio (i.e., limited exposure during baseline) as well as a smaller index BZD prescription—were both associated with elevated OD risk. A potential mechanism is that limited exposure may be less safe due to heightened sensitivity to the sedating effects of the medication. These findings run counter to current recommendations for use [9, 12, 13] and suggest that, paradoxically, limiting supply may not be associated with protection from OD risk. It would be incorrect to conclude from these results that prescribing larger quantities is safer; however, our findings do demonstrate that those who have lower levels of exposure are at elevated OD risk.

Prior work examining BZD regimen and risk of injury has focused on fall-related injury among older adults dually enrolled in Medicare and Medicaid [20, 21]. Consistent with our OD findings, these investigators found, relative to non-use of BZD, increased risk of injury associated with higher dosages and within the first two weeks of the index prescription, but risk did not vary meaningfully by BZD type (e.g., long- vs. short-acting). Park et al.’s examination of OD risk for BZD-opioid co-prescribing provides additional useful context: as with fall-related injury, they found OD liability to be similar across specific BZDs [35]. Therefore, given limited differentiation of injury liability among individual BZD medications, we did not examine them separately. However, consistent with prior work among Medicare beneficiaries [36], it is notable that alprazolam was the most widely prescribed overall, which is unfortunate because it is the most misused BZD [31].

Numerous groups have demonstrated the increased risk of opioid-related OD among persons also consuming BZD [33, 34, 37], so we expected to find concurrent opioid use associated with increased risk of OD. However, current antipsychotic use was also associated with a significantly increased risk of OD across all four cohorts, with effect sizes nearly as large as those of opioids. With the exception of antidepressants among those in the incident BZD cohorts, concurrent exposure to all the other medication classes examined were associated with an increased risk of OD across both age groups of incident and continuing BZD users.

Our study has several limitations. This observational work allows us to examine factors associated with elevated risk but does not establish causality; removing the factors associated with elevated risk may not reduce OD risk. The association of lower OD risk with smaller medication supply may reflect unobserved confounding, with clinicians more cautiously prescribing to patients whom they perceive to be at high risk. While our study included key observed characteristics potentially associated with OD risk, our measures of medication exposure are based on prescription claims and may not reflect actual consumption. While our study accounts for the presence of a diagnosed substance use disorder, we could not capture co-ingestion of alcohol or other substances along with their BZD. Finally, the study is limited to those with Part D prescription drug coverage and does not include those in Medicare Advantage, which limits the generalizability.

## Conclusions

While clinicians continue to prescribe BZD, there is relatively little information to help guide safer prescribing, with the exception of co-prescribing with opioids or use in older adults or those with substance use disorders [38]. This evidence gap is particularly striking in light of the significant and growing toll of BZD-related OD and death [2, 4, 5, 39]. While our research does not establish causality, we do identify characteristics of the baseline pattern of BZD use, index BZD prescription, and other medications that may be useful features for clinicians to consider as they try to prescribe safely. Perhaps most notably, we found that low levels of BZD exposure—both of the index BZD as well as during the 6-month baseline for continuing users—were associated with elevated OD risk. This study suggests important next steps for further investigation to isolate causal effects, focusing specifically on different patterns of BZD exposure and co-prescribing.

## Supplementary Information

**Additional file 1:** **Figure S1.** Flow Chart of the Study Population Examining Factors Associated with Overdose Events among Incident and Continuing Benzodiazepine Users. **Table S1.** Medications Contributing to Each Medication Class. **Figure S2.** Computing Medication Possession Ratio for Continuing Benzodiazepine Users. **Table S2.** Types of Overdose Event by Cohort. **Table S3.** Unadjusted Characteristics Associated with Overdose Event Among Incident and Continuing Benzodiazepine Users Stratified by Age. **Table S4.** Adjusted Characteristics Associated with Overdose Event Among Incident and Continuing Benzodiazepine Users Stratified by Age.

## Data Availability

The data that support the findings of this study are available from the Centers for Medicare & Medicaid Services (CMS) but restrictions apply to the availability of these data, which were used under a data use agreement for the current study, and so are not publicly available. Investigators can independently request the data used to construct this cohort from CMS.

## Abbreviations

BZD: Benzodiazepine
OD: Overdose
aHR: Adjusted hazard ratio
U.S: United States of America
ICD-10-CM: *International Classification of Diseases, 10* ^*th*^ *Revision–Clinical Modification*
CDC: Centers for Disease Control and Prevention
Lor-eq mg: Lorazepam-equivalent milligrams
MPR: Medication possession ratio
Z-drugs: Non-benzodiazepine benzodiazepine receptor agonists
LR: Log-rank
SD: Standard deviation
CI: Confidence interval
