# Supplementary material for: Prescription characteristics associated with drug overdose risk among adults prescribed benzodiazepines: a cohort study
Source: BMC Pharmacol Toxicol. 2023 May 19;24:34. doi: 10.1186/s40360-023-00674-x (PMC10199543; doi:10.1186/s40360-023-00674-x)
Supplement: Supplementary file 1 — Additional file 1: Figure S1. Flow Chart of the Study Population Examining Factors Associated with Overdose Events among Incident and Continuing Benzodiazepine Users. Table S1. Medications Contributing to Each Medication Class. Figure S2. Computing Medication Possession Ratio for Continuing Benzodiazepine Users. Table S2. Types of Overdose Event by Cohort. Table S3. Unadjusted Characteristics Associated with Overdose Event Among Incident and Continuing Benzodiazepine Users Stratified by Age. Table S4. Adjusted Characteristics Associated with Overdose Event Among Incident and Continuing Benzodiazepine Users Stratified by Age. [file 40360_2023_674_MOESM1_ESM.docx]

### **Supplementary Material**

### **Figure S1.** Flow Chart of the Study Population Examining Factors Associated with Overdose Events among Incident and Continuing Benzodiazepine Users

## **Table S1.** Medications Contributing to Each Medication Class

**Figure S2.** Computing Medication Possession Ratio (MPR) for Continuing Benzodiazepine Users

**Table S2.** Types of Overdose Event by Cohort

## **Table S3.** Unadjusted Characteristics Associated with Overdose Event Among Incident and Continuing Benzodiazepine Users Stratified by Age

## **Table S4.** Adjusted Characteristics Associated with Overdose Event Among Incident and Continuing Benzodiazepine Users Stratified by Age

### **Figure S1.** Flow Chart of the Study Population Examining Factors Associated with Overdose Events among Incident and Continuing Benzodiazepine Users

**B. Continuing Cohort**

**A. Incident Cohort**

**3,994,494** excluded

**3,994,494** no BZD fill between 4/1/16 and 12/31/17 during 6+ month period of continuous coverage

**146,436** excluded

**146,436** did not have a BZD fill preceded by 6 months of continuous coverage before

**16,669** had an OD event in the previous 6 months

**1,176,351** with at least one BZD filled between 4/1/16 and 12/31/17 during 6+ month period of continuous coverage

**5,170,845** alive on 4/1/16, resided in 50 states/DC, with 6+ months continuous fee-for-service and Part D coverage between 10/1/15 and 12/31/17^a^

**1,029,915** with at least one BZD fill preceded by 6 months continuous coverage

**250,254** excluded

**250,254** no BZD fill in prior 6 months

**748,588** continuing BZD users with no missing data

**756,364** alive on day of continuing BZD use, no poisoning events in prior 6 months

**779,661** at least one BZD fill and 1+ BZD fill in prior 6 months^b^

**7,776** excluded

**7,776** missing race or rurality

**23,297** excluded

**47** died before day of continuing BZD use

**23,250** had poisoning event in prior 6 months

**3,994,494** excluded

**3,994,494** no BZD fill between 4/1/16 and 12/31/17 during 6+ month period of continuous coverage

**146,436** excluded

**146,436** did not have a BZD fill preceded by 6 months of continuous coverage before

**16,669** had an OD event in the previous 6 months

**516,088** excluded

**516,088** had BZD fill in prior 6 months

**15,786** excluded

**65** died before day of incident BZD use

**15,721** had poisoning event in prior 6 months

**6,353** excluded

**6,353** missing race or rurality

**5,170,845** alive on 4/1/16, resided in 50 states/DC, with 6+ months continuous fee-for-service and Part D coverage between 10/1/15 and 12/31/17^a^

**1,176,351** with at least one BZD filled between 4/1/16 and 12/31/17 during 6+ month period of continuous coverage

**1,029,915** with at least one BZD fill preceded by 6 months continuous coverage

**498,041** alive on day of incident BZD use, no poisoning events in prior 6 months

**513,827** at least one BZD fill and no BZD fill in prior 6 months^b^

**491,688** incident BZD users with no missing data

## BZD, benzodiazepine

## ^a^ Among a Medicare 20% sample. Continuous fee for-service means that a given beneficiary had continuous Parts A and B and no Part C coverage. Part A is hospital insurance and covers hospital stays including inpatient and skilled nursing stays. Part B is medical insurance covers services and equipment such as office visits and wheelchairs. Part C (or Medicare Advantage) means that a given beneficiary elects to obtain their Part A and B insurance coverage through a private health insurance company; those with coverage not through a private health insurance company are considered “fee-for-service” (or “traditional Medicare”). Part D is prescription drug coverage.

^b^ If multiple BZD fills met the inclusion criteria for a given beneficiary, the earliest was selected. The BZD filled (incident or continuing) is referred to as the index BZD prescription.

## **Table S1.** Medications Contributing to Each Medication Class

| **Medication class** | **AHFS Codes** | **Medications** |
| --- | --- | --- |
| Benzodiazepines | 28:24.08 | Alprazolam, Chlordiazepoxide, Clorazepate, Diazepam, Estazolam, Flurazepam, Halazepam, Lorazepam, Midazolam, Oxazepam, Prazepam, Quazepam, Temazepam, Triazolam, Clobazam, Clonazepam |
| Antidepressants | 28:16.04.xx | Isocarboxazid, Phenelzine, Tranylcypromine,Rasagiline, SelegilineDesvenlafaxine, Duloxetine, Levomilnacipran,Venlafaxine, MilnacipranCitalopram, Escitalopram, Fluoxetine,Fluvoxamine, Paroxetine, Sertraline, VilazodoneNefazodone, Trazodone, VortioxetineAmitriptyline, Amoxapine, Clomipramine,Desipramine, Doxepin, Imipramine, Maprotiline, Nortriptyline, Protriptyline, TrimipramineBupropion, Mirtazapine |
| Antiepileptics | 28.12.xx.xxexceptbenzodiazepines | Phenobarbital, Primidone, MethohexitalEthotoin, Fosphenytoin, PhenytoinEthadione, Paramethadione, TrimethadioneEthosuximide, MethsuximideBrivaracetam, Carbamazepine, Eslicarbazepine,Felbamate, Gabapentin, Lacosamide, Lamotrigine,Levetiracetam, Magnesium Sulfate, Oxcarbazepine,Perampanel, Pregabalin, Rufinamide, Sultiame, Tiagabine, Topiramate, Valproate/Divalproex/Valproic Acid, Vigabatrin, Zonisamide, Acetazolamide |
| Antipsychotics | 28:16.08.xx | Aripiprazole, Asenapine, Brexpiprazole,Cariprazine, Clozapine, Iloperidone, Lurasidone, Olanzapine, Paliperidone, Pimavanserin, Quetiapine, Risperidone, ZiprasidoneHaloperidolChlorpromazine, Fluphenazine, Perphenazine,Prochlorperazine, Thioridazine, TrifluoperazineThiothixeneLoxapine, Molindone, Pimozide |
| Opioids | 28:08.08 and28:08.12 | Codeine, Fentanyl, Hydrocodone, HydromorphoneLevorphanol, Meperidine, Methadone, Morphine, Opium, Oxycodone, Oxymorphone, Remifentanil, Sufentanil, Tapentadol, Tramadol, Buprenorphine, Butorphanol, Nalbuphine, Pentazocine, Dihydrocodeine |
| Z-drugs | Subset of 28:24.92 | Eszopiclone, Zaleplon, Zolpidem |

AHFS, American Hospital Formulary Service.

**Figure S2.** Computing Medication Possession Ratio (MPR) for Continuing Benzodiazepine Users^a^


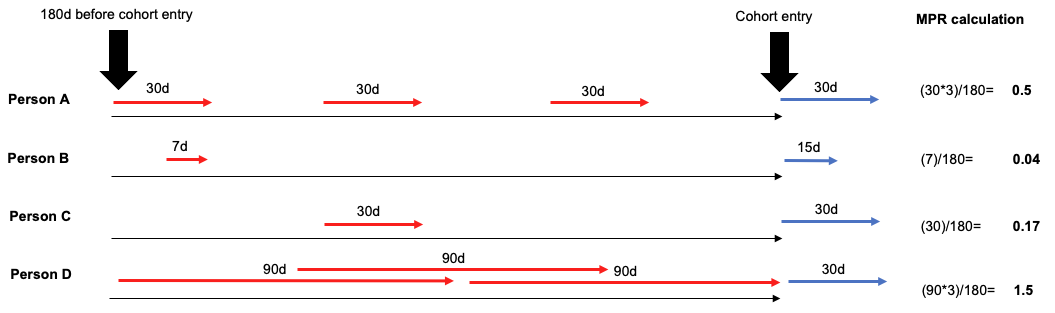


MPR, medication possession ratio; d, days’ supply.

^a^ Time of cohort entry for each person is indicated by the blue arrows (i.e., index BZD fill). Red arrows indicate BZD prescription fills during the 6-month baseline prior to cohort entry. MPR was calculated based by summing these baseline BZD fill dates and dividing by 180.

**Table S2.** Types of Overdose Event by Cohort

|  | **Incident^b^** | | **Continuing** | |
| --- | --- | --- | --- | --- |
| ***ICD-10-CM* T Code**^a^ | **<65**  **N=1,058** | **65+**  **N=2,843** | **<65**  **N=1,619** | **65+**  **N=2,609** |
| T36  *systemic antibiotics* | 41 (3.9) | 132 (4.6) | 61 (3.8) | 120 (4.6) |
| T37 *other systemic anti-infectives and antiparasitics* | 12 (1.1) | 47 (1.7) | 28 (1.7) | 48 (1.8) |
| T38 *hormones and their synthetic substitutes* | 145 (13.7) | 405 (14.2) | 175 (10.8) | 360 (13.8) |
| T39 *nonopioid analgesics, antipyretics, and antirheumatics* | 39 (3.7) | 72 (2.5) | 67 (4.1) | 88 (3.4) |
| T40  *narcotics and psychodysleptics* | 248 (23.4) | 449 (15.8) | 440 (27.2) | 436 (16.7) |
| T41  *anesthetics and therapeutic gases* | 6 (0.6) | 21 (0.7) | 6 (0.4) | 16 (0.6) |
| T42  *antiepileptic, sedative-hypnotic and antiparkinsonism drugs* | 240 (22.7) | 403 (14.2) | 425 (26.3) | 376 (14.4) |
| T42.4X  *benzodiazepines* | 173 (16.4) | 277 (9.7) | 295 (18.2) | 234 (9.0) |
| T43  *psychotropic drugs, not elsewhere classified* | 123 (11.6) | 159 (5.6) | 168 (10.4) | 154 (5.9) |
| T44  *drugs primarily affecting the autonomic nervous system* | 24 (2.3) | 87 (3.1) | 41 (2.5) | 94 (3.6) |
| T45  *primarily systemic and hematological agents, not elsewhere classified* | 146 (13.8) | 764 (26.9) | 179 (11.1) | 586 (22.5) |
| T46  *agents primarily affecting the cardiovascular system* | 41 (3.9) | 168 (5.9) | 71 (4.4) | 171 (6.6) |
| T47  *agents primarily affecting the gastrointestinal system* | 6 (0.6) | 19 (0.7) | 6 (0.4) | 20 (0.8) |
| T48  *agents primarily acting on smooth and skeletal muscles and the respiratory system* | 11 (1.0) | 18 (0.6) | 22 (1.4) | 23 (0.9) |
| T49  *topical agents primarily affecting skin and mucous membrane and by ophthalmological, otorhinorlaryngological and dental drugs* | 4 (0.4) | 9 (0.3) | 5 (0.3) | 6 (0.2) |
| T50  *diuretics and other and unspecified drugs, medicaments and biological substances* | 148 (14.0) | 445 (15.7) | 224 (13.8) | 432 (16.6) |

^a^ Includes codes with a 6^th^ character (signifying intent) of 1 (accidental), 2 (intentional), 4 (undetermined), or 5 (adverse effect)—e.g., T42.4X4 (overdose by benzodiazepine of undetermined intent)—with select exceptions where these are the 5^th^ character, as outlined by Vivolo-Kantor et al.^[[1]](#footnote-2)^

^b^ The percentages for each cohort may not sum to 100 as there can be multiple T-codes listed per encounter.

## **Table S3.** Unadjusted Characteristics Associated with Overdose Event Among Incident and Continuing Benzodiazepine Users Stratified by Age^a^

|  | **HR (95% CI)** | | | |
| --- | --- | --- | --- | --- |
|  | **Benzodiazepine Use** | | | |
|  | **Incident** | | **Continuing** | |
| **Characteristic** | **<65** | **65+** | **<65** | **65+** |
| *Sociodemographics* |  |  |  |  |
| Sex |  |  |  |  |
| Male | 1 (ref) | 1 (ref) | 1 (ref) | 1 (ref) |
| Female | 0.86 (0.77-0.97)* | 0.69 (0.64-0.74)*** | 1.12 (1.01-1.24)* | 0.84 (0.78-0.91)*** |
| Age (categorized) |  |  |  |  |
| <45 | 1 (ref) | - | 1 (ref) | - |
| 45-64 | 0.97 (0.85-1.11) | - | 0.98 (0.87-1.1) | - |
| 65-74 | - | 1 (ref) | - | 1 (ref) |
| 75-84 | - | 1.12 (1.04-1.21)** | - | 1.11 (1.02-1.21)* |
| 85+ | - | 1.09 (0.99-1.2) | - | 1.02 (0.91-1.14) |
| Race^a^ |  |  |  |  |
| Non-Hispanic White | 1 (ref) | 1 (ref) | 1 (ref) | 1 (ref) |
| Non-Hispanic Black | 1 (0.85-1.18) | 1.22 (1.05-1.41)** | 1.16 (1.01-1.34)* | 1.17 (0.99-1.38) |
| Hispanic | 0.98 (0.8-1.21) | 1.09 (0.94-1.27) | 0.96 (0.8-1.16) | 1.04 (0.88-1.22) |
| Asian/Pacific Islander | 1.18 (0.71-1.94) | 0.84 (0.64-1.1) | 1.06 (0.65-1.75) | 0.92 (0.67-1.28) |
| Other | 0.77 (0.46-1.3) | 1.03 (0.71-1.49) | 0.97 (0.64-1.49) | 0.66 (0.39-1.11) |
| Low-income subsidy^b^ |  |  |  |  |
| No | 1 (ref) | 1 (ref) | 1 (ref) | 1 (ref) |
| Yes | 1.52 (1.27-1.82)*** | 1.28 (1.18-1.38)*** | 1.56 (1.35-1.82)*** | 1.29 (1.2-1.4)*** |
| Rurality^c^ |  |  |  |  |
| Urban | 1 (ref) | 1 (ref) | 1 (ref) | 1 (ref) |
| Rural | 0.94 (0.79-1.11) | 1.04 (0.94-1.16) | 0.81 (0.71-0.94)** | 0.98 (0.88-1.09) |
| *Clinical Characteristics* |  |  |  |  |
| Frailty |  |  |  |  |
| Not frail | - | 1 (ref) | - | 1 (ref) |
| Frail | - | 2.63 (2.45-2.82)*** | - | 3.31 (3.06-3.58)*** |
| Elixhauser |  |  |  |  |
| 0-1 | 1 (ref) | 1 (ref) | 1 (ref) | 1 (ref) |
| 2 | 1.67 (1.35-2.07)*** | 1.95 (1.65-2.31)*** | 1.46 (1.22-1.75)*** | 1.44 (1.2-1.72)*** |
| 3 | 1.57 (1.25-1.98)*** | 2.6 (2.21-3.06)*** | 1.86 (1.55-2.23)*** | 2.26 (1.91-2.68)*** |
| 4 | 2.31 (1.84-2.89)*** | 3.65 (3.11-4.28)*** | 2.22 (1.84-2.69)*** | 2.86 (2.41-3.39)*** |
| 5 | 3.05 (2.42-3.83)*** | 4.72 (4.02-5.55)*** | 2.58 (2.1-3.16)*** | 3.94 (3.32-4.67)*** |
| 6 | 3.37 (2.62-4.32)*** | 6.12 (5.2-7.2)*** | 4.51 (3.72-5.48)*** | 5.6 (4.72-6.63)*** |
| 7 | 3.29 (2.48-4.36)*** | 7.16 (6.04-8.49)*** | 5.12 (4.14-6.34)*** | 6.26 (5.22-7.5)*** |
| 8 | 3.9 (2.88-5.29)*** | 8.34 (6.99-9.96)*** | 6.65 (5.3-8.32)*** | 8.26 (6.86-9.95)*** |
| 9+ | 7.1 (5.82-8.65)*** | 12.23 (10.58-14.14)*** | 9.35 (7.89-11.08)*** | 12.06 (10.34-14.06)*** |
| Substance Use Disorder |  |  |  |  |
| Not present | 1 (ref) | 1 (ref) | 1 (ref) | 1 (ref) |
| Present | 2.43 (2.16-2.73)*** | 2.09 (1.89-2.3)*** | 2.25 (2.04-2.47)*** | 2.3 (2.09-2.53)*** |
| Personality Disorder |  |  |  |  |
| Not present | 1 (ref) | 1 (ref) | 1 (ref) | 1 (ref) |
| Present | 2.09 (1.62-2.71)*** | 1.11 (0.61-2.02) | 2.17 (1.78-2.64)*** | 1.96 (1.33-2.9)*** |
| *BZD Characteristics* |  |  |  |  |
| Medication possession ratio |  |  |  |  |
| <0.5 | - | - | 1.37 (1.24-1.52)*** | 1.41 (1.29-1.54)*** |
| 0.5-1 | - | - | 1 (ref) | 1 (ref) |
| >1 | - | - | 1.28 (1.06-1.55)** | 1.42 (1.15-1.75)** |
| Index average daily dose, mg |  |  |  |  |
| <1 | 1 (ref) | 1 (ref) | 1 (ref) | 1 (ref) |
| 1-1.99 | 1.24 (1.03-1.48)* | 1.49 (1.36-1.63)*** | 1.26 (1.03-1.53)* | 1.23 (1.11-1.37)*** |
| 2+ | 1.49 (1.26-1.78)*** | 1.63 (1.48-1.79)*** | 1.28 (1.07-1.54)** | 1.29 (1.16-1.43)*** |
| Index supply, days |  |  |  |  |
| <14 | 1.20 (1.06-1.35)** | 1.35 (1.26-1.45)*** | 1.67 (1.47-1.91)*** | 1.89 (1.72-2.07)*** |
| 14-30 | 1 (ref) | 1 (ref) | 1 (ref) | 1 (ref) |
| 31+ | 1.15 (0.84-1.57) | 0.46 (0.38-0.56)*** | 0.65 (0.52-0.82)*** | 0.65 (0.57-0.74)*** |
| *Other Medication Use* |  |  |  |  |
| Antidepressants |  |  |  |  |
| Never | 1 (ref) | 1 (ref) | 1 (ref) | 1 (ref) |
| Former | 1.55 (1.29-1.87)*** | 1.29 (1.13-1.46)*** | 1.59 (1.36-1.85)*** | 1.58 (1.40-1.79)*** |
| Current | 1.12 (0.99-1.27) | 1.05 (0.97-1.13) | 1.14 (1.02-1.27)* | 1.25 (1.15-1.35)*** |
| Antiepileptics |  |  |  |  |
| Never | 1 (ref) | 1 (ref) | 1 (ref) | 1 (ref) |
| Former | 1.73 (1.43-2.08)*** | 1.73 (1.52-1.96)*** | 1.82 (1.57-2.12)*** | 1.71 (1.49-1.95)*** |
| Current | 1.48 (1.31-1.68)*** | 1.68 (1.55-1.83)*** | 1.55 (1.40-1.72)*** | 1.83 (1.68-1.99)*** |
| Antipsychotics |  |  |  |  |
| Never | 1 (ref) | 1 (ref) | 1 (ref) | 1 (ref) |
| Former | 1.73 (1.38-2.17)*** | 3.43 (3.00-3.93)*** | 1.91 (1.62-2.24)*** | 3.21 (2.81-3.67)*** |
| Current | 1.41 (1.23-1.61)*** | 2.50 (2.26-2.76)*** | 1.23 (1.10-1.37)*** | 1.76 (1.57-1.96)*** |
| Opioids |  |  |  |  |
| Never | 1 (ref) | 1 (ref) | 1 (ref) | 1 (ref) |
| Former | 1.47 (1.25-1.74)*** | 1.92 (1.75-2.10)*** | 1.98 (1.72-2.27)*** | 1.82 (1.66-2.01)*** |
| Current | 1.75 (1.53-1.99)*** | 2.65 (2.45-2.86)*** | 1.92 (1.71-2.16)*** | 2.48 (2.28-2.71)*** |
| Z-drugs |  |  |  |  |
| Never | 1 (ref) | 1 (ref) | 1 (ref) | 1 (ref) |
| Former | 1.43 (1.06-1.92)* | 1.00 (0.81-1.23) | 1.81 (1.49-2.20)*** | 1.13 (0.93-1.36) |
| Current | 1.27 (1.02-1.57)* | 1.22 (1.04-1.42)* | 1.34 (1.15-1.55)*** | 1.19 (1.01-1.39)* |

*<.05; **<.01; ***<.001

HR, hazard ratio; CI, confidence interval; BZD, benzodiazepine.

^a^ Derived using the Research Triangle Institute race variable; race groups are mutually exclusive.

^b^ Considered present if a given beneficiary was enrolled or eligible in the Part D low-income subsidy for at least one month during the 6-month baseline period.

^c^ Derived using beneficiary state and county codes and Rural-Urban Continuum Codes.

## **Table S4.** Adjusted Characteristics Associated with Overdose Event Among Incident and Continuing Benzodiazepine Users Stratified by Age^a^

|  | **HR (95% CI)** | | | |
| --- | --- | --- | --- | --- |
|  | **Benzodiazepine Use** | | | |
|  | **Incident** | | **Continuing** | |
| **Characteristic** | **<65** | **65+** | **<65** | **65+** |
| *Sociodemographics* |  |  |  |  |
| Sex |  |  |  |  |
| Male | 1 (ref) | 1 (ref) | 1 (ref) | 1 (ref) |
| Female | 0.92 (0.81-1.04) | 0.85 (0.79-0.91)*** | 1.06 (0.95-1.17) | 0.94 (0.87-1.02) |
| Age (categorized) |  |  |  |  |
| <45 | 1 (ref) | - | 1 (ref) | - |
| 45-64 | 0.81 (0.70-0.93)** | - | 0.85 (0.76-0.96)** | - |
| 65-74 | - | 1 (ref) | - | 1 (ref) |
| 75-84 | - | 0.94 (0.87-1.02) | - | 1 (0.91-1.09) |
| 85+ | - | 0.78 (0.70-0.86)*** | - | 0.85 (0.76-0.96)** |
| Race^b^ |  |  |  |  |
| Non-Hispanic White | 1 (ref) | 1 (ref) | 1 (ref) | 1 (ref) |
| Non-Hispanic Black | 0.82 (0.69-0.97)* | 0.91 (0.78-1.06) | 0.86 (0.74-0.99)* | 0.87 (0.74-1.04) |
| Hispanic | 0.94 (0.77-1.16) | 1.09 (0.93-1.27) | 0.85 (0.70-1.03) | 0.98 (0.83-1.16) |
| Asian/Pacific Islander | 1.34 (0.81-2.21) | 0.99 (0.75-1.31) | 1.18 (0.72-1.95) | 1.06 (0.76-1.48) |
| Other | 0.71 (0.42-1.20) | 0.92 (0.63-1.33) | 0.92 (0.60-1.40) | 0.58 (0.35-0.99)* |
| Low-income subsidy^c^ |  |  |  |  |
| No | 1 (ref) | 1 (ref) | 1 (ref) | 1 (ref) |
| Yes | 1.24 (1.03-1.50)* | 0.75 (0.68-0.81)*** | 1.27 (1.09-1.48)** | 0.78 (0.72-0.86)*** |
| Rurality^d^ |  |  |  |  |
| Urban | 1 (ref) | 1 (ref) | 1 (ref) | 1 (ref) |
| Rural | 0.94 (0.79-1.12) | 1.01 (0.91-1.11) | 0.84 (0.73-0.97)* | 0.96 (0.86-1.07) |
| *Clinical Characteristics* |  |  |  |  |
| Frailty |  |  |  |  |
| Not frail | - | 1 (ref) | - | 1 (ref) |
| Frail | - | 1.07 (0.97-1.17) | - | 1.40 (1.26-1.55)*** |
| Elixhauser |  |  |  |  |
| 0-1 | 1 (ref) | 1 (ref) | 1 (ref) | 1 (ref) |
| 2 | 1.58 (1.28-1.96)*** | 1.89 (1.60-2.23)*** | 1.37 (1.14-1.65)*** | 1.33 (1.11-1.6)** |
| 3 | 1.45 (1.15-1.83)** | 2.42 (2.05-2.85)*** | 1.69 (1.40-2.03)*** | 1.94 (1.63-2.30)*** |
| 4 | 2.08 (1.65-2.62)*** | 3.27 (2.78-3.86)*** | 1.94 (1.60-2.35)*** | 2.25 (1.89-2.68)*** |
| 5 | 2.68 (2.12-3.39)*** | 4.04 (3.41-4.78)*** | 2.17 (1.76-2.67)*** | 2.84 (2.37-3.4)*** |
| 6 | 2.90 (2.24-3.74)*** | 5.07 (4.26-6.03)*** | 3.67 (3.01-4.47)*** | 3.75 (3.12-4.51)*** |
| 7 | 2.78 (2.09-3.72)*** | 5.73 (4.77-6.88)*** | 4.04 (3.24-5.02)*** | 3.96 (3.25-4.82)*** |
| 8 | 3.19 (2.34-4.36)*** | 6.49 (5.35-7.86)*** | 5.07 (4.02-6.39)*** | 4.90 (3.99-6.01)*** |
| 9+ | 5.74 (4.64-7.09)*** | 9.06 (7.66-10.71)*** | 6.86 (5.73-8.22)*** | 6.58 (5.5-7.87)*** |
| Substance Use Disorder |  |  |  |  |
| Not present | 1 (ref) | 1 (ref) | 1 (ref) | 1 (ref) |
| Present | 1.73 (1.53-1.96)*** | 1.19 (1.08-1.32)*** | 1.50 (1.35-1.66)*** | 1.38 (1.25-1.53)*** |
| Personality Disorder |  |  |  |  |
| Not present | 1 (ref) | 1 (ref) | 1 (ref) | 1 (ref) |
| Present | 1.33 (1.02-1.74)* | 0.48 (0.26-0.88)* | 1.43 (1.16-1.75)*** | 1.01 (0.68-1.49) |
| *BZD Characteristics* |  |  |  |  |
| Medication possession ratio |  |  |  |  |
| <0.5 | - | - | 1.20 (1.06-1.36)** | 1.12 (1.01-1.24)* |
| 0.5-1 | - | - | 1 (ref) | 1 (ref) |
| >1 | - | - | 1.18 (0.98-1.43) | 1.19 (0.96-1.47) |
| Index average daily dose, mg |  |  |  |  |
| <1 | 1 (ref) | 1 (ref) | 1 (ref) | 1 (ref) |
| 1-1.99 | 1.19 (0.99-1.43) | 1.33 (1.22-1.46)*** | 1.25 (1.03-1.53)* | 1.17 (1.05-1.3)** |
| 2+ | 1.40 (1.17-1.66)*** | 1.28 (1.16-1.41)*** | 1.35 (1.12-1.63)** | 1.22 (1.09-1.36)*** |
| Index supply, days |  |  |  |  |
| <14 | 1.16 (1.03-1.31)* | 1.21 (1.13-1.30)*** | 1.33 (1.15-1.53)*** | 1.43 (1.30-1.57)*** |
| 14-30 | 1 (ref) | 1 (ref) | 1 (ref) | 1 (ref) |
| 31+ | 1.36 (0.99-1.85) | 0.56 (0.46-0.67)*** | 0.74 (0.59-0.94)* | 0.79 (0.69-0.90)*** |
| *Other Medication Use* |  |  |  |  |
| Antidepressants |  |  |  |  |
| Never | 1 (ref) | 1 (ref) | 1 (ref) | 1 (ref) |
| Former | 1.15 (0.95-1.39) | 0.87 (0.77-0.99)* | 1.18 (1.01-1.38)* | 1.15 (1.01-1.30)* |
| Current | 0.94 (0.82-1.08) | 0.82 (0.76-0.89)*** | 0.96 (0.86-1.08) | 0.96 (0.89-1.05) |
| Antiepileptics |  |  |  |  |
| Never | 1 (ref) | 1 (ref) | 1 (ref) | 1 (ref) |
| Former | 1.21 (1.00-1.46) | 1.10 (0.96-1.25) | 1.24 (1.07-1.45)** | 1.07 (0.93-1.23) |
| Current | 1.14 (1.002-1.30)* | 1.10 (1.01-1.20)* | 1.20 (1.08-1.34)** | 1.18 (1.08-1.30)*** |
| Antipsychotics |  |  |  |  |
| Never | 1 (ref) | 1 (ref) | 1 (ref) | 1 (ref) |
| Former | 1.30 (1.03-1.65)* | 2.25 (1.96-2.58)*** | 1.46 (1.24-1.73)*** | 1.99 (1.73-2.27)*** |
| Current | 1.24 (1.07-1.44)** | 1.99 (1.79-2.21)*** | 1.20 (1.07-1.35)** | 1.33 (1.18-1.50)*** |
| Opioids |  |  |  |  |
| Never | 1 (ref) | 1 (ref) | 1 (ref) | 1 (ref) |
| Former | 1.11 (0.94-1.32) | 1.40 (1.27-1.53)*** | 1.43 (1.24-1.65)*** | 1.31 (1.19-1.44)*** |
| Current | 1.36 (1.18-1.57)*** | 1.80 (1.66-1.96)*** | 1.49 (1.31-1.70)*** | 1.73 (1.58-1.90)*** |
| Z-drugs |  |  |  |  |
| Never | 1 (ref) | 1 (ref) | 1 (ref) | 1 (ref) |
| Former | 1.17 (0.87-1.57) | 0.93 (0.76-1.15) | 1.36 (1.12-1.66)** | 0.99 (0.82-1.19) |
| Current | 1.16 (0.94-1.44) | 1.20 (1.03-1.41)* | 1.21 (1.04-1.41)* | 1.13 (0.96-1.33) |

*<.05; **<.01; ***<.001

HR, hazard ratio; CI, confidence interval; BZD, benzodiazepine.

^a^ Each column presents results from a separate Cox proportional hazards model for each of the four cohorts. All four models include all row characteristics as well as seasonality by including month of the index BZD prescription as a categorical variable.

^b^ Derived using the Research Triangle Institute race variable; race groups are mutually exclusive.

^c^ Considered present if a given beneficiary was enrolled or eligible in the Part D low-income subsidy for at least one month during the 6-month baseline period.

^d^ Derived using beneficiary state and county codes and Rural-Urban Continuum Codes.

1. Vivolo-Kantor A, Pasalic E, Liu S, Martinez PD, Gladden RM, Overdose Morbidity Team. Defining indicators for drug overdose emergency department visits and hospitalisations in *ICD-10-CM* coded discharge data. *Inj Prev* 2021;**27**:i56–61. [↑](#footnote-ref-2)
